# Clearance of bacteria from lymph nodes in sheep immunized with *Brucella suis* S2 vaccine is associated with M1 macrophage activation

**DOI:** 10.1186/s13567-023-01147-z

**Published:** 2023-03-14

**Authors:** Si Chen, Yuanyuan Chen, Zizhuo Jiao, Chengqiang Wang, Dantong Zhao, Yongbin Liu, Wenguang Zhang, Shihua Zhao, Bin Yang, Qinan Zhao, Shaoyin Fu, Xiaolong He, Qiaoling Chen, Churiga Man, Guoying Liu, Xuefeng Wei, Li Du, Fengyang Wang

**Affiliations:** 1 Hainan Key Lab of Tropical Animal Reproduction, Breeding and Epidemic Disease Research, Animal Genetic Engineering Key Lab of Haikou, School of Animal Science and Technology, Hainan University, Haikou, Hainan China; 2 Jinyu Baoling Bio-Pharmaceutical Co., Ltd., Hohhot, Inner Mongolia China; 3 Inner Mongolia University, College Road No. 235, Hohhot, Inner Mongolia China; 4 College of Life Science, Inner Mongolia Agricultural University, Hohhot, Inner Mongolia China; 5 Inner Mongolia Academy of Agriculture and Animal Husbandry Sciences, Hohhot, Inner Mongolia China

**Keywords:** *Brucella*, sheep, vaccine, lymphatic nodes, macrophages

## Abstract

Ovine brucellosis is a global zoonotic disease of sheep caused by *Brucella melitensis*, which inflicts a significant burden on human and animal health. *Brucella suis* strain S2 (B. suis S2) is a smooth live attenuated vaccine for the prevention of ovine brucellosis in China. However, no previous studies have assessed the immunogenicity of B. suis S2 vaccine after oral immunization in sheep. Here, we attempted to evaluate the ovine immune response over the course of B. suis S2 immunization and to identify in vivo predictors for vaccine development. Body temperature, serum *Brucella* antibodies, serum cytokines (IL-12p70 and interferon [IFN]-γ), and bacterial load in the mandibular lymph nodes (LN), superficial cervical LN, superficial inguinal LN, and spleen were investigated to determine the safety and efficacy of the vaccine. The abnormal body temperature of sheep occurred within 8 days post-infection (dpi). *Brucella suis* S2 persisted for a short time (< 21 dpi) in the mandibular LN. The highest level of IL-12p70 was observed at 9 dpi, whereas serum IFN-γ levels peaked at 12 dpi. Transcriptome analysis and quantitative reverse transcription PCR were performed to determine gene expression profiles in the mandibular LN of sheep. Antigen processing and presentation pathway was the dominant pathway related to the dataset. Our studies suggest that the immune response in ovine LN resembled type 1 immunity with the secretion of IL-12p70 and IFN-γ after B.suis S2 immunization and the vaccine may eliminate *Brucella* via stimulation of M1 macrophages through the course of Th cells.

**Supplementary Information:**

The online version contains supplementary material available at 10.1186/s13567-023-01147-z.

## Introduction

Brucellosis is a zoonotic disease with an annual global incidence rate of approximately half a million human cases [1]. In China, *Brucella melitensis* is the primary etiological agent of human brucellosis and is transmitted to humans via exposure to infected sheep and goats or consumption of contaminated meat products. Therefore, eradicating infections in herds is crucial for preventing human contagion [2]. Abortion and infertility are the predominant clinical signs in small ruminants. Preventive measures, including culling of infected animals and vaccination of healthy animals, have resulted in the effective control and eradication of brucellosis. Currently, the reported vaccines against *Brucella* in sheep and goats include live-attenuated vaccines [3], genetically engineered attenuated vaccines [4, 5], vector-delivered *Brucella* vaccines [6–8], subunit vaccines [9] and others [10]. Despite several promising results, the efficacy and performance of these vaccines have not yet been systematically studied. The immunogenicity and principal characteristics of brucellosis need to be evaluated before its use in the herd.

The current live-attenuated vaccines have various drawbacks, including interference with diagnostic tests, induction of abortions in pregnant sheep, persistent infection, virulence in humans and risk of virulence reversion [11]. In China, the B. suis strain S2 vaccine is designed to prevent brucellosis in sheep and goats via the oral route in drinking water, which does not lead to abortion in pregnant females [12]. *Brucella suis* S2 is a live attenuated vaccine that was first isolated from the fetus of aborted swine in 1952 and spontaneously attenuated [13]. The advantages of the B. suis S2 vaccine are its low virulence, broad applicability, and convenient administration [14]. Animal experiments have shown that it protects mice from a virulent challenge by *B. melitensis* M28, *B. abortus* 2308 and B. suis S1330, and the S2 vaccinated mice did not develop any clinical signs or tissue damage [15]. A systematic analysis of laboratory brucellosis infection and vaccine infection events from 2006 to 2019 in China suggested that the S2 vaccine strain is virulent for humans because of the fatigue and sweat seen in infected individuals [16]. We believe that further investigation of the S2 vaccine strain pathogenicity in humans is necessary. Presently, studies in sheep and goats have mainly focused on the mechanism of B. suis S2 immune escape at the cellular level, involving both phagocytic and non-phagocytic cells. Recent studies on goat alveolar macrophages have demonstrated that B. suis S2 manipulates host inflammatory responses by inhibiting TLR/NF-κB and attenuating NLRP3 inflammasome activation [17]. In goat trophoblast cells, B. suis S2 induces apoptosis through endoplasmic reticulum stress, thereby hampering cell proliferation [18]. In caprine endometrial epithelial cells, B.suis S2 induces non-apoptotic ER stress via the PERK pathway [19]. To the best of our knowledge, no studies have assessed the immune defense mechanisms of oral vaccination with B. suis S2 in sheep. Lack of in vivo data has restricted understanding of the vaccine’s pregnancy-sparing advantages. Thus, it is crucial to investigate the immunogenicity of the B. suis S2 vaccine and mechanisms of sheep immune defense in vivo.

Several studies have reported that the immune defense mechanisms induced by B. suis S2 vaccine against *Brucella* include the innate immune signaling pathway, cell adhesion pathway, and adaptive immune signaling pathway. In RAW264.7 cells stimulated with B. suis S2, the most upregulated genes were related to the innate immune signaling pathway after 24 h of infection, including cytokines (IL-1, IL-6, IL-23, and Cfs3) and chemokines (Ccl2, Ccl3, Ccl4, Ccl5, and Ccl10) [20]. In *cynomolgus* monkeys immunized with the S2 vaccine, 663 differentially expressed genes (DEG) were involved in various biological processes, including the chemokine signaling pathway, defense response, immune system processing, and type-I interferon signaling pathway [21]. In sheep, four significant pathways and nine candidate genes (*CTNNA3*, *PARD3*, *PTPRM*, *NLGN1*, *CNTNAP2*, *NCAM1*, *PRKG1*, *ADCY2* and *YAP1*) related to brucellosis susceptibility were identified by whole-genome resequencing [22]. Our previous study identified three novel miRNA (novel_229, novel_609, and novel_973) in the lymph nodes of sheep immunized with B. suis S2, which participated in innate immunity, adaptive immunity, defense responses to bacteria, and the Notch signaling pathways [23].

Macrophages constitute the first line of defense in the innate immune response against invading *Brucella* [24]. It has long been recognized that *Brucella* interaction with macrophages is a key aspect of immune escape. Macrophages can polarize into two distinct subsets: M1 and M2. M1 macrophages, also called classically activated macrophages, are polarized by lipopolysaccharide (LPS) and Th1 cytokines such as interferon (IFN)-γ [25]. Activated M1 macrophages produce pro-inflammatory cytokines that trigger an inflammatory response, phagocytosis, and cytotoxicity. Our in vitro study on mouse macrophages (RAW264.7 cells) demonstrated that TNF-α secretion, iNOS expression, and NO production are stimulated by *B. melitensis* M5-90 [26]. Previous studies have indicated that genes involved in NF-kappa B signaling pathway were found to be significantly increased in B. suis S2 infected RAW264.7 cells at 48 h post infection [27]. B. suis S2 and its derivatives induce marked expression of IL-1β, IL-6, and TNF-α mRNA in RAW264.7 cells [28].

In this study, we examined the immunogenicity and protective capacity of the B. suis S2 vaccine in sheep to understand the immune mechanisms underlying brucellosis resistance. Our results suggest that the B. suis S2 vaccine is safe for sheep, and immunization with B. suis S2 results in an altered expression of various genes related to antigen processing and the presentation pathway of the host, with M1 macrophage expression identified as the dominant surrogate of protection. The role of M1 macrophages was first demonstrated in vivo in sheep immunized with the B. suis S2 vaccine.

## Materials and methods

### Animals

Small-tail Han female sheep, aged 10 months, were purchased and transported to the Biosafety Level 3 (BSL-3) laboratory of Jinyu Baoling Bio-pharmaceutical Co., Ltd (Inner Mongolia, China) for experiment. Sheep were serologically tested using the Pourquier^®^ Rose Bengal Brucellosis Antigen (IDEXX, P00215, ME, USA) and AsurDx™ Brucella Multispecies Antibodies cELISA Test Kit (BIOSTONE, 10043-05, TX, USA), which were designed for the detection of antibodies specific to *B. abortus*, *B. melitensis* and B. suis in bovine, ovine, caprine or swine. All sheep were housed separately in the Biosafety Level 3 (BSL-3) laboratory with daily supplemental feeding and water ad libitum. Animals were randomly divided into three groups: the body temperature monitoring group (T group, *n* = 6), the control group (C group, *n* = 3) and the vaccinated group (inoculated with B. suis S2, *n* = 23). At the indicated time points post infection, sheep in the vaccinated group were slaughtered humanely and sterilized by autoclaving. The tissues were collected for further experiments.

### Oral immunization with B. suis S2 vaccine

The lyophilized B. suis S2 vaccine was purchased from Jinyu Baoling Bio-pharmaceutical Co., Ltd (Inner Mongolia, China). To examine its accuracy in counting live bacteria, the number of colony-forming units (CFU) were confirmed retrospectively by counts of distinct colonies of the tryptic soy agar (BD Biosciences, NJ, USA) at 37 °C for 3–5 days. In the control group, three sheep were inoculated with 1 mL sterilized phosphate-buffered saline (PBS, pH 7.2). The remaining sheep in the T group and the vaccinated group were immunized with the lyophilized B. suis S2 vaccine (with an amount of 2 × 10^10^ CFU in 1 mL sterilized PBS) via oral administration. The control group and vaccinated group were monitored at 0, 7, 14, 21, and 30 days post-immunization (dpi) individually.

### Body temperature monitoring and the rose bengal test assay

The body temperature of six sheep in the T group were measured continuously and recorded each morning from 0 to 30 dpi. One-way ANOVA followed by Dunnett multiple comparisons test was performed using GraphPad Prism Software (v8.0.0, San Diego, California, USA). Ovine serum samples in the vaccinated group were collected prior to the first immunization and at 7, 14, 21, and 30 dpi. Serum samples were tested using the Pourquier^®^ Rose Bengal Brucellosis Antigen (IDEXX, P00215, ME, USA). In the rose bengal test (RBT) assay, 25 µL of each serum was dispensed on the plates. The same volume of Rose Bengal Brucellosis Antigen was added beside each sample. The serum and Rose Bengal Brucellosis Antigen were mixed to produce a circle 2 cm in diameter. Any visible agglutination was interpreted as a positive test result.

### Bacterial counting in host peripheral immune organs

To evaluate bacterial counting in host peripheral immune organs, seven tissues of each sheep in the vaccinated group were collected at 7, 14, 21, and 30 dpi. At each time point, three sheep were selected. The collected tissues included right mandibular LN, left mandibular LN, right superficial cervical LN, left superficial cervical LN, right superficial inguinal LN, left superficial inguinal LN and spleen. The tissues were weighed, homogenized and serially diluted in sterile PBS. Dilutions were plated on the superior Farrell's medium (Oxoid, SR0083A, Basingstoke, UK) and incubated for 5 days at 37 °C. Plates were monitored daily for growth, and *Brucella* was identified based on morphological characteristics and PCR. The live bacterial were enumerated as mean CFU/g ± SD.

### Quantification of IL-12p70 and IFN-γ in serum

Three sheep in the vaccinated group were selected for serum cytokine detection. The serum concentrations of the pro-infammatory cytokines IL-12p70 and IFN-γ were examined via enzyme-linked immunosorbent assay (ELISA) on 96-well microplates. IL-12p70 was tested using RayBio^®^ Ovine IL-12p70 ELISA Kit (RayBiotech, ELO-IL12P70, GA, USA). The quantification of IFN-γ was tested using RayBio^®^ Ovine IFN-gamma ELISA Kit (RayBiotech, ELO-IFNg, GA, USA). In each well, 50 µL of serum was added to 50 µL of 1× diluent solution to obtain a 1:1 dilution following the instructions from the manufacturer. The OD value was read at 450 nm wavelength in a Multiskan^®^ FC reader (Thermo Scientific, Finland). Each serum was processed in triplicate. The results were analyzed using Sigma plot software (Version 14.0), with standard concentration on the x-axis (pg/mL) and absorbance on the y-axis.

### RNA sequencing

The bilateral mandibular LN of sheep in the vaccinated group were selected for the transcriptome analysis at 7, 14, 21, and 30 dpi. At each time point, three sheep were selected. Three micrograms of total RNA per mandibular LN were used to construct the RNA libraries. Sequencing libraries were generated using NEBNext^®^ Multiplex Small RNA Library Prep Set for Illumina^®^ (New England Biolabs, Beverly, MA, USA), and index codes were added to attribute sequences to each sample. The library quality was assessed on the Agilent Bioanalyzer 2100 system. The Illumina HiSeq^™^2500 was used for sequencing. The sequencing data were validated by a series of filtration steps. The valid data were mapped to the reference genome (GCA_000298735.1) using HISAT2 (Version 2.0.4) and they were used to assemble transcripts with the reference annotation by StringTie (Version 1.3.4d). Gene expression levels were normalized by fragments per kilobase of exon model per million mapped reads (FPKM). The FPKM calculation equation used was FPKM = cDNA fragments/Mapped Reads (Million) × Transcript Length (kb).

### Functional analyses of differentially expressed genes

The expression data for the 7 dpi, 14 dpi, 21 dpi, and 30 dpi groups were normalized to that of the C group. Differentially expressed genes (DEG) of two groups were analyzed using the DESeq R package (1.8.3). *P*-values were adjusted using the Benjamini-Hochberg method. A default corrected *P*-value of 0.05 and the |log_2_ (Fold change) | ≥ 1 were set as the threshold for significantly differential expressions. The Kyoto Encyclopedia of Genes and Genomes (KEGG) database and Gene Ontology (GO) databases were used for pathway annotation of the DEG.

### Quantitative reverse transcription PCR validation

The quantitative reverse transcription PCR (RT-qPCR) was used to verify the reliability of the RNA sequencing. Total RNA was transcribed into cDNA according to the manufacturer’s protocol from the M-MLV G III Frist-Strand Synthesis Kit (EB15012, Yugong Biolabs, Lianyungang). The qPCR was performed using RealUniversal PreMix (FP201, TIANGEN, Beijing) on an ABI 7500 Real-Time PCR System (Applied Biosystems, Foster City, CA, USA). The reference gene was *GAPDH*. Relative transcriptional levels were determined by the 2^−∆∆Ct^ method [29].

### Statistical analysis

The data were analyzed using the Mann-Whitney test, two-way or one-way analysis of variance (ANOVA), and Tukey multiple-comparison test as appropriate. GraphPad Prism software (La Jolla, CA, USA) was used for the analyses.

## Results

### Body temperature changed from 2 to 8 dpi

**Figure 1:**
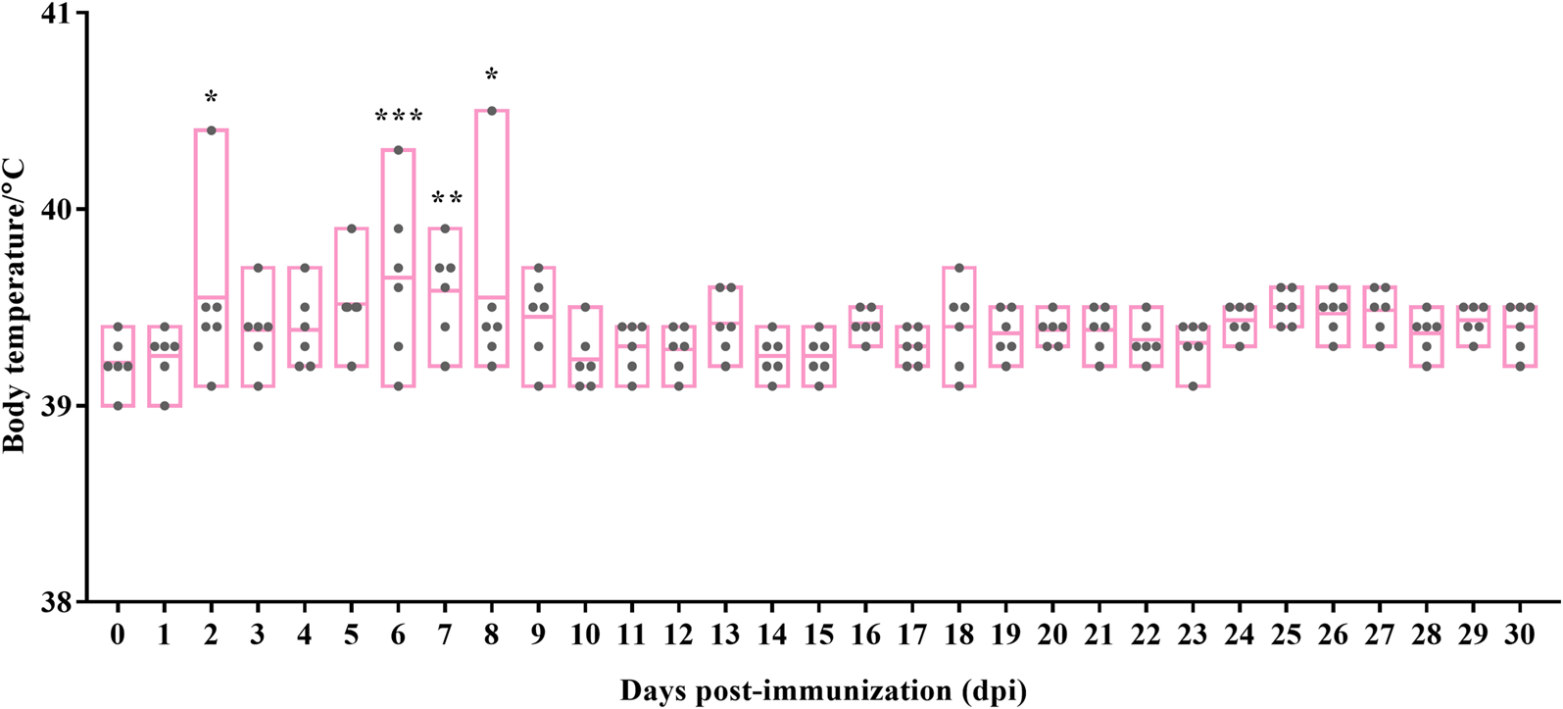
**The body temperature monitoring of six sheep in T group from 0 to 30 dpi.** *P < 0.05, **P < 0.01 and ***P < 0.001.

To evaluate the possible side effects of B. suis S2 vaccine, the body temperatures of six sheep in the T group were monitored continuously, starting from 1 day before immunization until 30 dpi. The clinical manifestations of B. suis S2 vaccine varied significantly among individual sheep. Some sheep exhibited classical undulant fever, while others remained asymptomatic. Statistical analysis indicated significantly higher temperatures and temperature fluctuations in individuals at 6 dpi (*P* < 0.001). From 2 to 8 dpi, B. suis S2 caused severe fluctuation in the body temperature of these sheep, ranging from 39.0 to 40.5 °C (Figure 1). At 8 dpi, all sheep body temperatures returned to the normal range. No other serious adverse reactions, such as chills and malaise, were observed in the vaccinated groups.

### *Brucella* antibodies in the serum persist up to at least 30 dpi

For B. suis serological antibody detection, ovine sera were collected and tested using the rose bengal test (RBT) at 7, 14, 21 and 30 dpi (Table 1). *Brucella* antibodies in the serum were detectable at 7 dpi. The *Brucella* seroprevalence measured by RBT, reached 100% at 21 dpi. At 30^th^ dpi, five out of six sheep in the vaccinated groups remained positive for RBT. At 120 days after vaccination, all vaccinated ewes tested negative in the RBT test.

**Table 1:** **The RBT assay results after vaccination.**

Days post-immunization (dpi)	RBP results		Positive rate (%)
	Number of positive animals	Number of negative animals	
7 dpi	1	4	20
14 dpi	5	1	83
21 dpi	6	0	100
30 dpi	5	1	83

### *Brucella suis* S2 only exists in mandibular LN and were eradicated at 21 dpi

To determine bacterial virulence in vivo, the survival of B. suis S2 in the peripheral immune organs of sheep was examined by determining the number of CFU. No B. suis S2 was isolated from the other tissues at any time point, except for the mandibular LN. According to the bacterial isolation results, B. suis S2 was randomly distributed in the left and right mandibular LN of the sheep (Table 2). Bacteria migrated to and persisted in the mandibular LN for at least 2 weeks. The number of B. suis S2 began to decline at two weeks and was completely eradicated after three weeks. In addition, all LN and spleens were unremarkable, with no evidence of lesions in the vaccinated groups at necropsy. However, despite the unremarkable gross appearance of the LN, histological sections of the mandibular LN show that B. suis S2 induced infiltration of inflammatory cells, such as macrophages (Additional file 1).

**Table 2:** **The colony forming units (CFU) of **B. suis **S2 isolates in sheep mandibular LN (mean CFU/g ± SD).**

Sheep	dpi	Left Mandibular LN (CFU/g)	Right Mandibular LN (CFU/g)
No.1	7	0	0
No.2		70 ± 80	0
No.3		1667 ± 208	4039 ± 449
No.4	14	0	429 ± 189
No.5		978 ± 278	0
No.6		0	332 ± 110
No.7	21	0	0
No.8		0	0
No.9		0	0
No.10	30	0	0
No.11		0	0
No.12		0	0

### Serum Th-1 type cytokine rises early at 9 dpi

**Figure 2:**
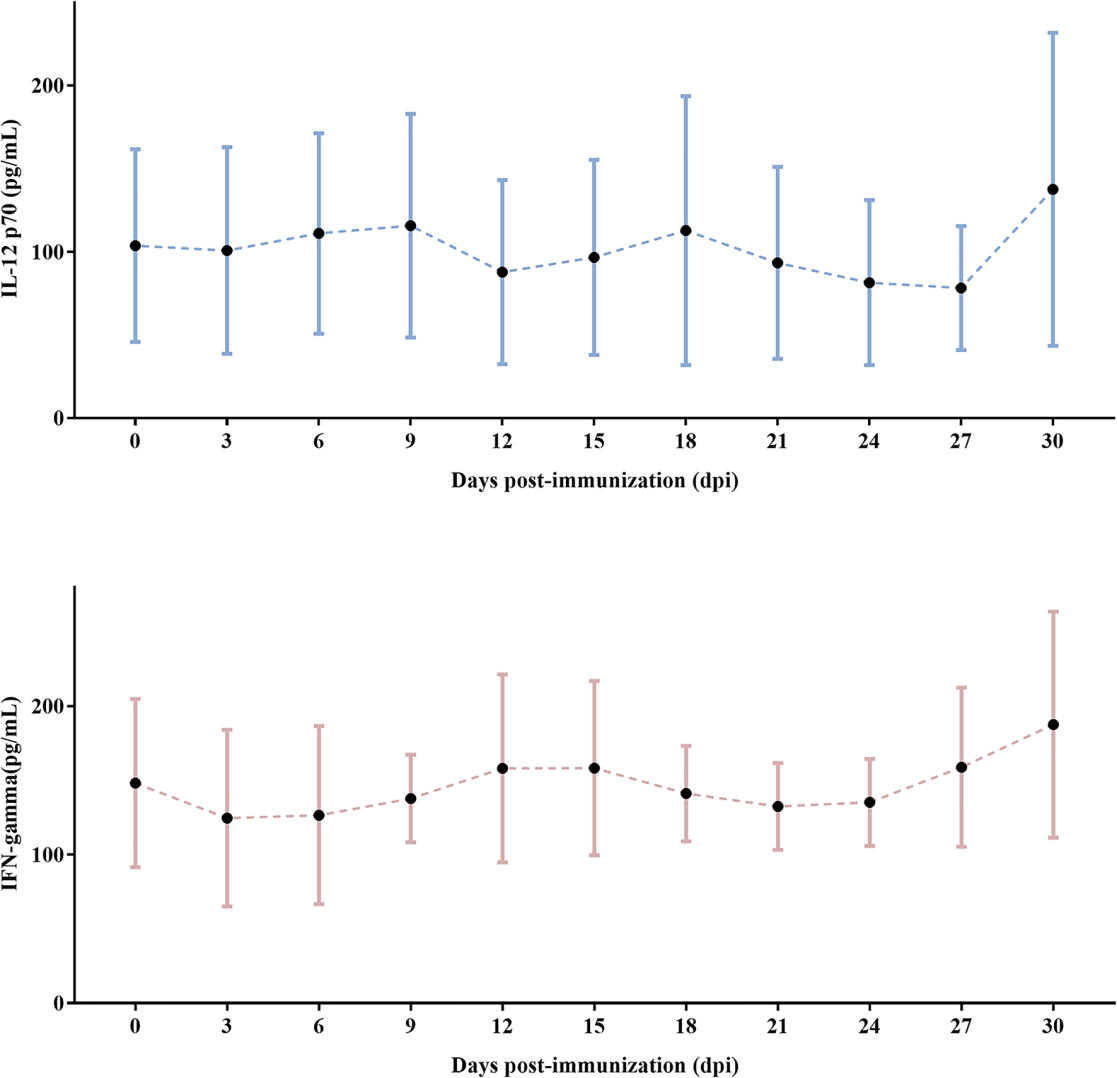
**The serum IL-12p70 and IFN-γ monitoring of four sheep from 0 to 30 dpi.**

IL-12p70 is required to boost Th-1 responses and IFN-γ production. IFN-γ can be induced by IL-12p70 independently during early infection and contributes to the innate immune response. The highest level of IL-12p70 was detected in the serum 9 days after immunization. This was accompanied by significantly higher serum IFN-γ levels at 12 dpi. The general trend in serum IL-12p70 level was undulating. IFN-γ peaked by day 12 to 15 after the first immunization and increased steadily from 18 to 30 dpi. These results demonstrate that IFN-γ maintained a response to the B. suis S2 vaccine. IFN-γ was produced at later time points after IL-12p70 stimulation (Figure 2).

### The chemotactic activity of T-lymphocytes is initiated at 7 dpi

**Figure 3:**
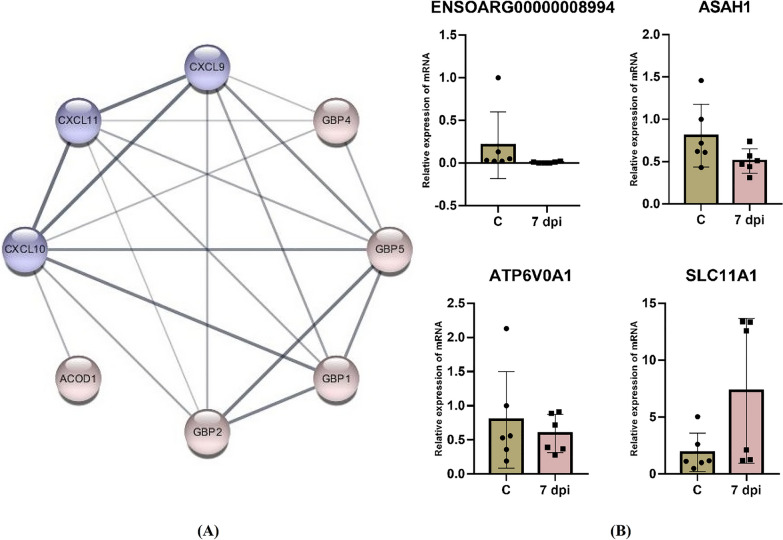
**Network of upregulated DEG and RT-qPCR results.** **A** Network of DEG associated with the cellular response to IFN-γ and Th1 cells migration. **B** The results of RT-qPCR verification.

Compared with the C group, the upregulated DEG in the vaccinated groups at 7 dpi were mainly associated with the defense response to bacteria and cellular response to IFN-γ. *LBP* and *ACOD1*, which belong to the toll-like receptor signaling pathway, were induced. The downstream genes of the toll-like receptor signaling pathway, *CXCL9*, *CXCL10* and *CXCL11*, which encoded IFN-inducible chemokines, were strikingly expressed (Figure 3A). These three genes are cell markers of pro-inflammatory M1 macrophages and prompt the chemotactic activity of Th1 cells [30]. *CCL28*, which encoded the mucosa-associated epithelial chemokine CCL28, were also unregulated. Meanwhile, *GBP1*, *GBP2*, *GBP4*, *GBP5*, and *GBP6*, which were members of the IFN-γ-inducible GTPase superfamily, were significantly activated. In contrast, six marker genes of natural killer cells (NK cells), including *NKG2A*, *KIR3DX1*, *CD94*, *ENSOARG00000002418*, *ENSOARG00000002450*, and *ENSOARG00000007834*, were all downregulated. Four genes involved in these pathways were selected for the RT-qPCR analysis. Among these, *ENSOARG00000008994*, *ATP6V0A1*, and *SLC11A1* were in accordance with the RNA-seq results (Figure 3B).

### The differentiation of CD4^+^ T cells is activated at 14 dpi

**Figure 4:**
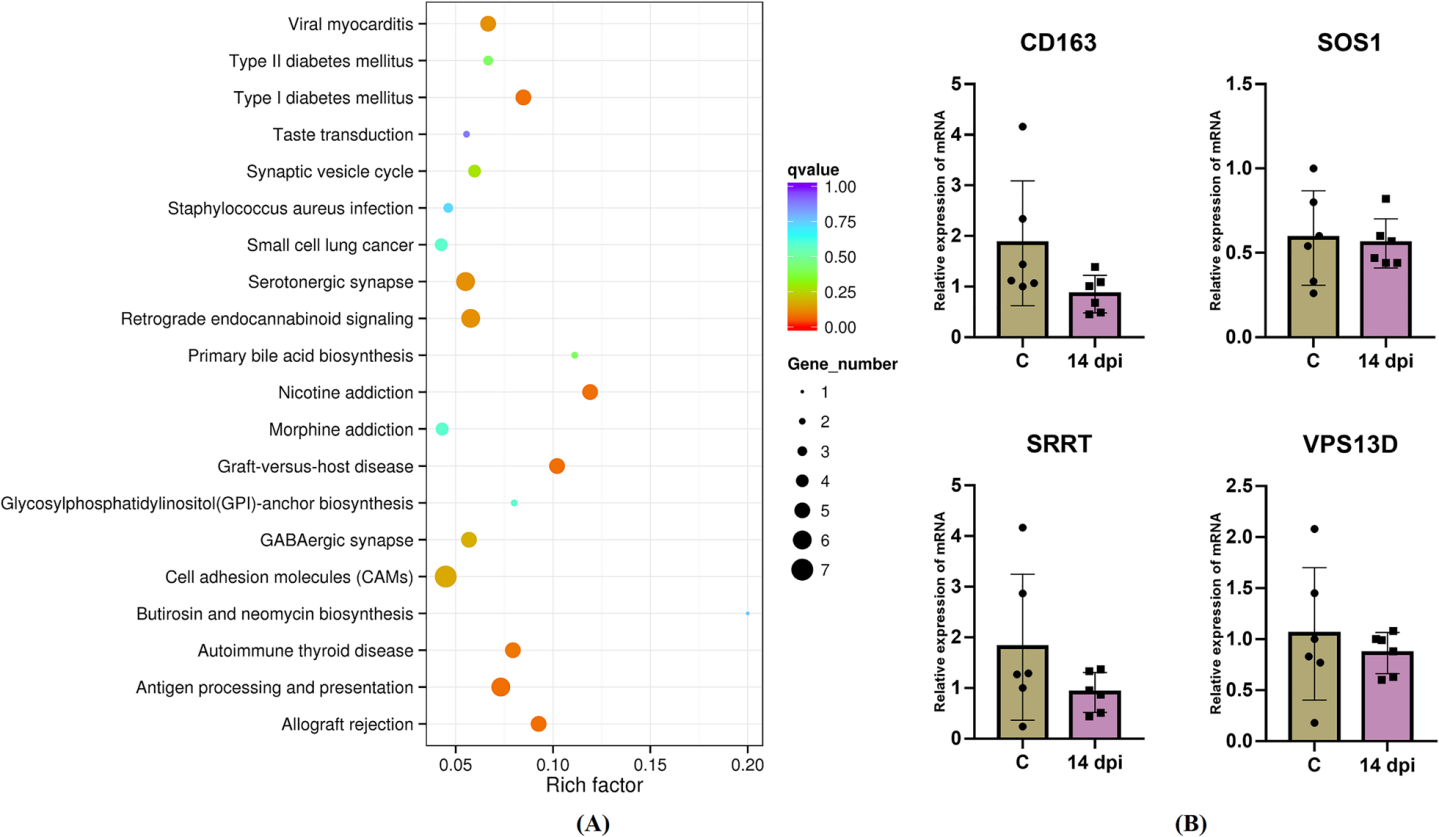
**KEGG pathway enrichment analysis of DEG and RT-qPCR results.** **A** KEGG pathway enrichment analysis of DEG (Top 20, FDR < 0.05). **B** The results of RT-qPCR verification.

Compared with the C group, the DEG in the 14 dpi vaccinated groups were prominently enriched in antigen processing and presentation and graft-versus-host disease pathways (Figure 4A). The M2 macrophage-specific gene, *CD163,* was downregulated from 14 to 30 dpi. *DRB3*, which belonged to the MHC class II gene family, was significantly increased at 14 dpi. The major histocompatibility complex (MHC) is a cluster of genes, most of which are responsible for presenting antigens to the immune system and playing a central role in regulating immune responses. MHC class II genes encode glycoproteins that bind to and present extracellular pathogens to circulating helper T lymphocytes and initiate cell-mediated immunity. Moreover, *ENSOARG00000002102*, *ENSOARG00000002532*, *ENSOARG00000002875*, and *ENSOARG00000014493*, which are known as MHC class I-like antigen recognition superfamily genes, were downregulated at 14 dpi. A total of three genes (*SOS1*, *SRRT,* and *VPS13D*) were tested by RT-qPCR analysis. This provided consistent results with the RNA-seq data (Figure 4B).

### The phagocytosis of macrophages enhanced at 21 dpi

**Figure 5:**
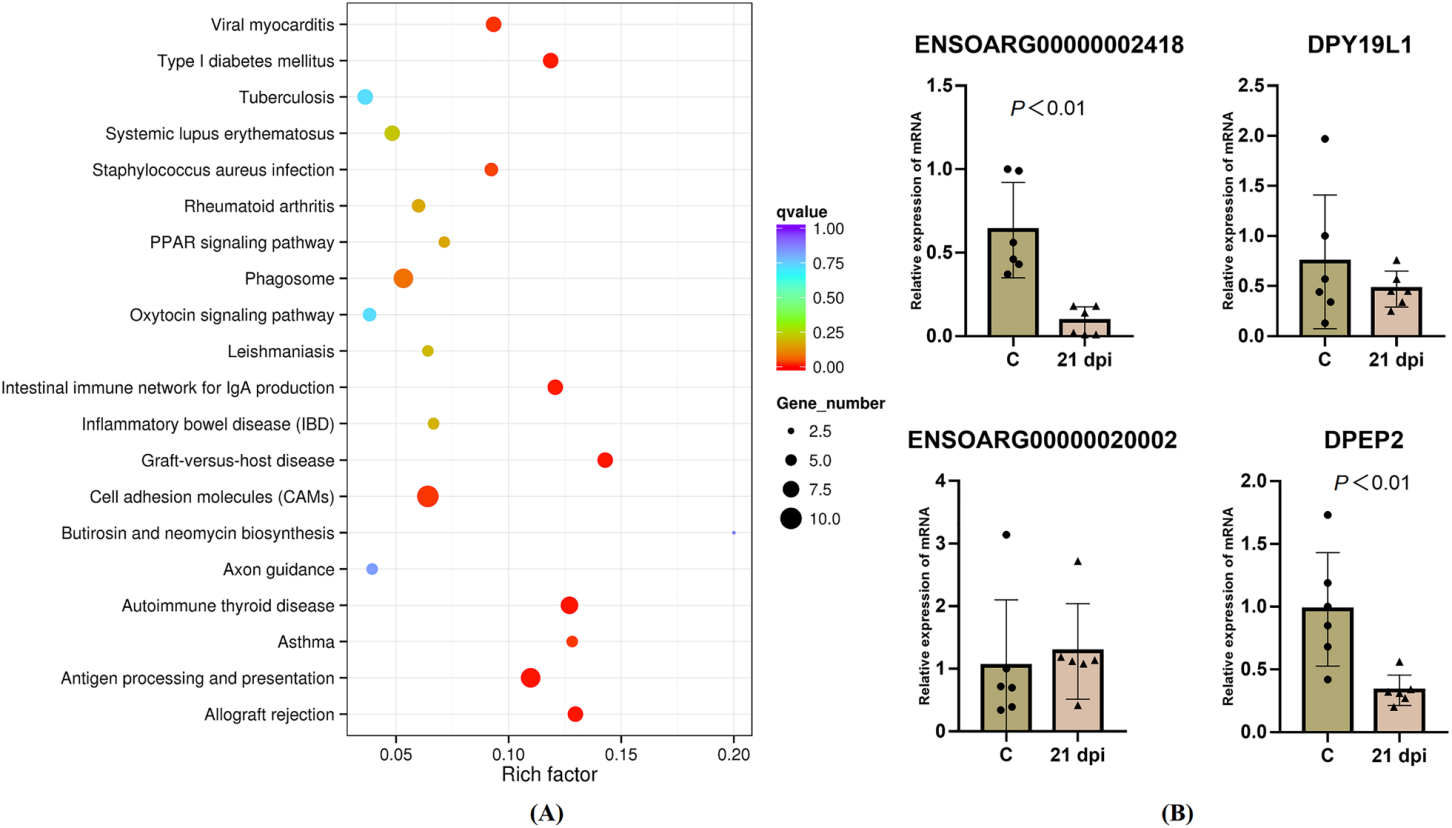
**KEGG pathway enrichment analysis of DEG and RT-qPCR results.** **A** KEGG pathway enrichment analysis of DEG (Top 20, FDR < 0.05). **B** The results of RT-qPCR verification.

The phagosome pathway was observed at the 21^st^ dpi (Figure 5A). *DYA*, *DQB1*, *PIKFYVE*, and *ENSOARG00000001181* were up-regulated, whereas *ENSOARG00000009357*, *DR1B*, *DPB1*, *MRC1*, and *ATP6V0A1* were down-regulated. Among these DEG, *PIKFYVE* plays an essential role in the maturation of early endosomes into late endosomes, phagosomes, and lysosomes. The *DYA* gene, which also belongs to the MHC class II gene family, is unique in both ovine and bovine species [31]. *MRC1* encodes the mannose receptor of macrophages and DC, which promotes bacterial uptake into phagosomes [32]. Decreased *MRC1* expression indicates a shift towards a pro-inflammatory environment and a switch from tolerogenic to immunogenic immune cell phenotypes, which enhances lysosomal fusion to avoid the intracellular niche for B. suis S2. The representative genes (*ENSOARG00000002418*, *DPEP2*, and *ENSOARG00000020002*) in the enrichment pathways were further verified by RT-qPCR, the results of which were consistent with the RNA-seq data (Figure 5B).

### Antigen processing and presentation is maintained up to 30 dpi

**Figure 6:**
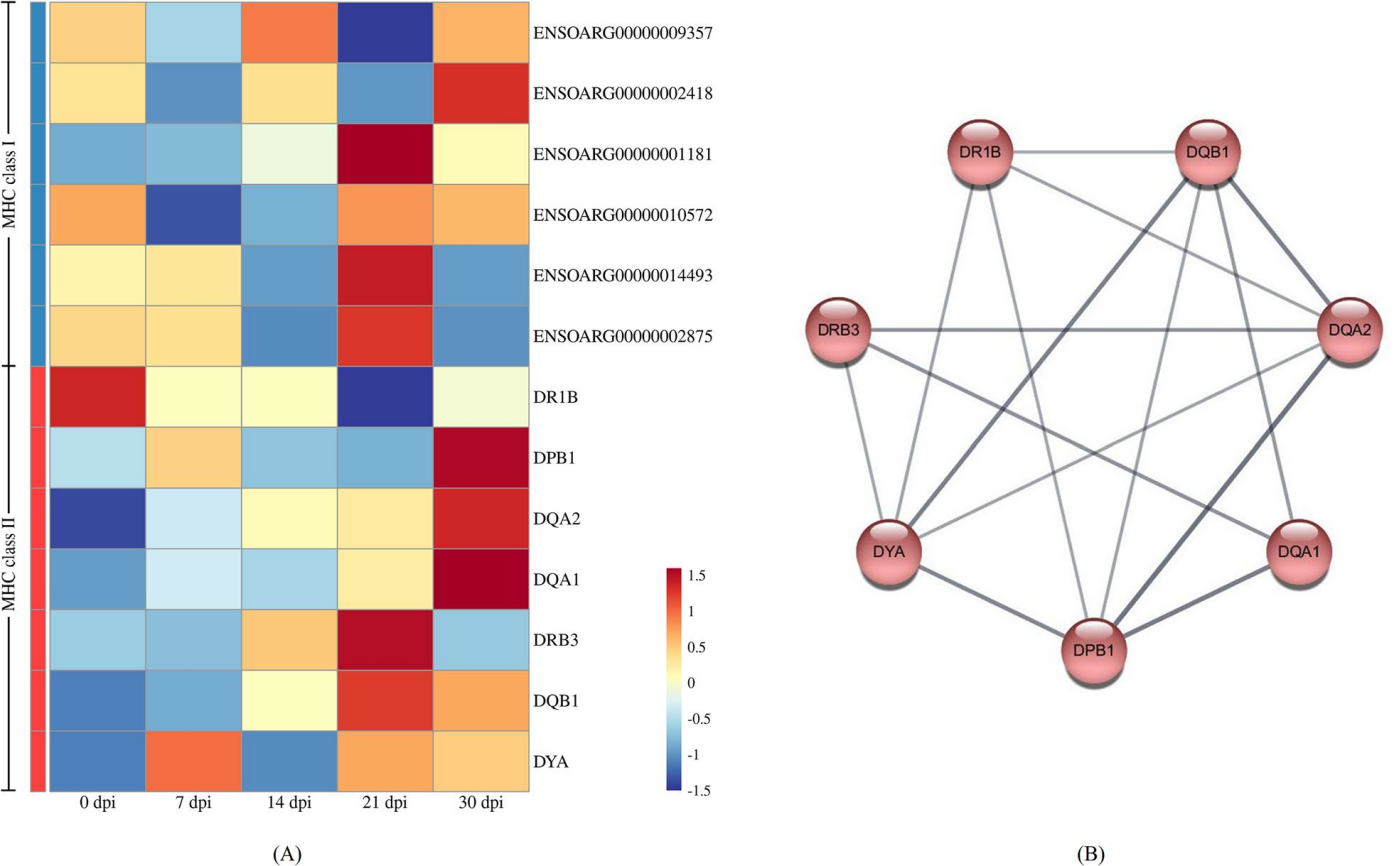
**The heatmap and regulatory networks of target DEG in antigen processing and presentation.** **A** Heatmap of DEG in antigen processing and presentation pathway. **B** The regulatory networks of DEG. Red plots represent the MHC class II protein complex.

Both the MHC class I and MHC class II protein complexes were identified from 7 to 30 dpi, which revealed that B. suis S2 in the mandibular LN were eradicated through these pathways (Figure 6A). According to the KEGG pathway enrichment analysis of DEG, the antigen processing and presentation pathway was activated at 30 dpi, including *DPB1*, *DYA*, *DQB1*, *DQA1* and *DQA2* genes (Figure 6B). Moreover, this pathway was detected at 7 dpi, suggesting that the adaptive cell-mediated immune response was initiated early. *ENSOARG00000002875* and *ENSOARG00000014493*, which belonged to the MHC class I-like antigen recognition-like superfamily, were downregulated at 30 dpi.

## Discussion

*Brucella* eludes innate immune recognition through modifications of its virulence factors, such as lipopolysaccharide (LPS) and flagellin, resulting in a mild pro-inflammatory response that leads to bacterial persistence. B. suis S2 occurs as a smooth strain, expressing smooth lipopolysaccharide as the major surface antigen [12], which is similar to *B. melitensis*. The predominant route of *B. melitensis* infection under natural exposure is the alimentary tract [33], which is also similar to the B. suis S2 vaccine. Thus, B.suis S2 is an ideal model for studying host immune response after *Brucella* infection. In this study, we demonstrate for the first time the ovine immune response, via the gastrointestinal mucosa routes, to B. suis S2 administration and evaluated the safety and protective efficacy of the B. suis S2 vaccine in vivo. Analysis of sheep anal temperature demonstrates that immunization of B. suis S2 led to an abnormal change in the body temperature of sheep within one week after immunization.

To induce immunity, live vaccine strains need to reach, multiply, and persist in immune-reactive tissues for a sufficient time, particularly in the spleen and/or lymph nodes. The survival time of B.suis S2 depends on residual virulence, dose, and route of administration. Studies by Wang showed that the murine spleen enlargements are detected following B. suis S2 infection (1 × 10^7^ CFU) after 14 days and are fully restored 28 days post-infection. Compared to uninfected mouse spleens, the number of macrophages increases significantly in the red pulp of spleens of B.suis S2-infected mice after 1 week [34]. According to our results, B. suis S2 were isolated as early as 7 dpi from the mandibular LN of the immunized sheep. The oral mucosa was the initial site of vaccination, and B.suis S2 were isolated from the mandibular LN. However, the spleen of sheep were clean, which was inconsistent with the murine model. At 21 dpi, *Brucella* clearance from the mandibular LN was observed, indicating that B. suis S2 persisted for a short time in the host. In addition, all vaccinated ewes were negative for the RBT test after four months. Therefore, a booster vaccination after the first administration may be necessary to induce long-time protection.

The present literature on B.suis S2 is largely based on the murine model, and in vivo data from sheep are limited. During B.suis S2 immunization in our ovine model, the host immune response resembled Th1 immunity with the secretion of IL-12p70 and IFN-γ. In the beginning of *B. abortus* infection, it has been clearly demonstrated that murine NK cells are the most important IFN-γ producers [35]. Human NK cells are activated by autologous infected macrophages and secrete TNF-α and IFN-γ, thereby controlling intramacrophagic development of B. suis in humans [36]. In our study, six marker genes of NK cells were downregulated at 7 dpi, indicating that the activity of NK cells in sheep mandibular LN was probably suppressed by B.suis S2. Despite the inhibition of NK cells observed at 7 dpi, downstream IFN-γ pathways were still activated by other immune cells. It is well known that the activation of pro-inflammatory M1 macrophages can be induced by the cytokine IFN-γ and bacterial components such as lipopolysaccharide (LPS) [37]. On the one hand, IFN-γ initiates the GBP superfamily promoting oxidative killing [38]. On the other hand, IFN-γ stimulates macrophages producing IL-12, a critical cytokine that evokes an adaptive immune response of type 1 helper T cells (Th1) and supports the continuous production of IFN-γ [39]. In our study, the highest level of IL-12p70 was detected in the serum 9 days after immunization. In addition, activated M1 macrophages produced high levels of pro-inflammatory cytokines such as CXCL9, CXCL10, and CXCL11, at 7 dpi, leading to the migration of T cells to inflamed tissue sites along chemokine gradients.

To mediate pathogen clearance, the Th0 cells are activated by IL-12p70 and are transformed into Th1 cells. Animal studies have demonstrated that adequate Th1 immunity, with significant production of IFN-γ and IL-12, is the principal immune effector for the clearance of *Brucella* infection. According to the ELISA results, the IFN-γ levels in the serum peaked at 12–15 dpi, reflecting the massive activation of Th1 cells. IFN-γ plays an essential role in bidirectional stimulation of T cells and macrophages. By producing large amounts of IFN-γ and IL-12, Th1 cells induce the activation and M1 polarization of macrophages and enhance macrophage function, cell cytotoxic and Th1 proliferation [40]. In the present study, M1 macrophages probably eliminated B. suis S2 by enhancing phagocytosis and expressing *DRB3*. DRB3 belongs to the MHC class II gene family and is responsible for presenting antigens to T cells. A number of studies have reported the association of BoLA-DRB3.2 alleles with susceptibility/resistance to some infectious diseases in cattle, such as bovine papillomavirus infection [41]. Th1-driven IFN-γ enhances phagocytosis of M1 macrophages to kill intracellular microorganisms. *PIKFYVE*, which expressed at 21 dpi, participated in the generation of Stage I melanosomes and maintenance of ion homeostasis in lysosomes [42]. Moreover, DYA, which comprised the MHC class II molecules of macrophages, was significantly upregulated from 21 to 30 dpi.

Under IFN-γ stimulation, the MHC class II molecules of M1 macrophages, *DPB1*, *DQA1*, and *DQA2* genes, were expressed at 30 dpi. The antigen presentation ability of macrophages was enhanced. Prolonged inflammation leads to tissue damage. The body also produces M2 macrophages with anti-inflammatory activities. Classical monocytes diminish *CD163* levels on the membrane and preferentially acquire *CD163*^−^ defined M1 characteristics upon in vitro LPS stimulation [43]. CD163 is a high-affinity scavenger receptor that is typically associated with the M2 macrophage phenotype [44]. From lymph nodes in sheep immunized with B. suis S2 vaccine, we detected a low level of *CD163* expression sustained from 14 to 30 dpi. Whether it was regulated by LPS of B. suis S2 or host IFN-γ, needs to be further investigated. Moreover, CCL28, which belongs to the CC chemokine (β-chemokine) signaling family, is first observed in the lymph nodes of sheep immunized with B. suis S2 throughout the process. CCL28 plays dual roles in the regulation of mucosal immune responses and recruitment of T cells into nasal mucosal tissues [45]. In this study, high CCL28 expression levels provide a constitutive innate immune defense against various bacterial pathogens driving mucosal homing of T and B lymphocytes with the above chemokines. In mice, sublingual immunization can effectively protect against *Helicobacter pylori* infection by enhancing CXCL10 and CCL28, resulting in strong T and B cell infiltration into the stomach [46].

In conclusion, our study suggests that B. suis S2 vaccinated sheep may control infection via stimulation of M1 macrophages through the course of Th cells. Briefly, the polarization of M1 macrophages was induced by B. suis S2 LPS or IFN-γ as early as 7 dpi. With the activation of Th1 immunity, Th1-driven IFN-γ enhanced phagocytosis of M1 macrophages at 14 dpi. Thus, M1 macrophages eventually eliminated the intracellular microorganisms at 21 dpi. Our study has gained insight into in vivo observations regarding the critical role of M1 macrophages in the control of B. suis S2 infections. The mechanism by which B. suis S2 influences host infection is complex, and our study provides evidence for one possible mechanism. Further experiments are required to confirm these findings. We are currently exploring new approaches to investigate genetic markers with natural resistance to *Brucella* infection, which can be further used in marker-assisted selection for natural resistance to brucellosis in breeding programs, as a significant contribution to the prevention of the disease in small ruminant herds.

## Supplementary Information

**Additional file 1. The histopathology of mandibular lymph nodes at different times. A** 0 dpi; **B** 7 dpi. (H&E, 5×).

## Data Availability

The raw sequence data reported in this paper have been deposited in the Genome Sequence Archive [47] in National Genomics Data Center [48], China National Center for Bioinformation / Beijing Institute of Genomics, Chinese Academy of Sciences (GSA: CRA007560) that are publicly accessible at https://ngdc.cncb.ac.cn/gsa.
