# Supplementary figures and images for: Clearance of bacteria from lymph nodes in sheep immunized with Brucella suis S2 vaccine is associated with M1 macrophage activation
Source: Vet Res. 2023 Mar 14;54:20. doi: 10.1186/s13567-023-01147-z (PMC10013293; doi:10.1186/s13567-023-01147-z)

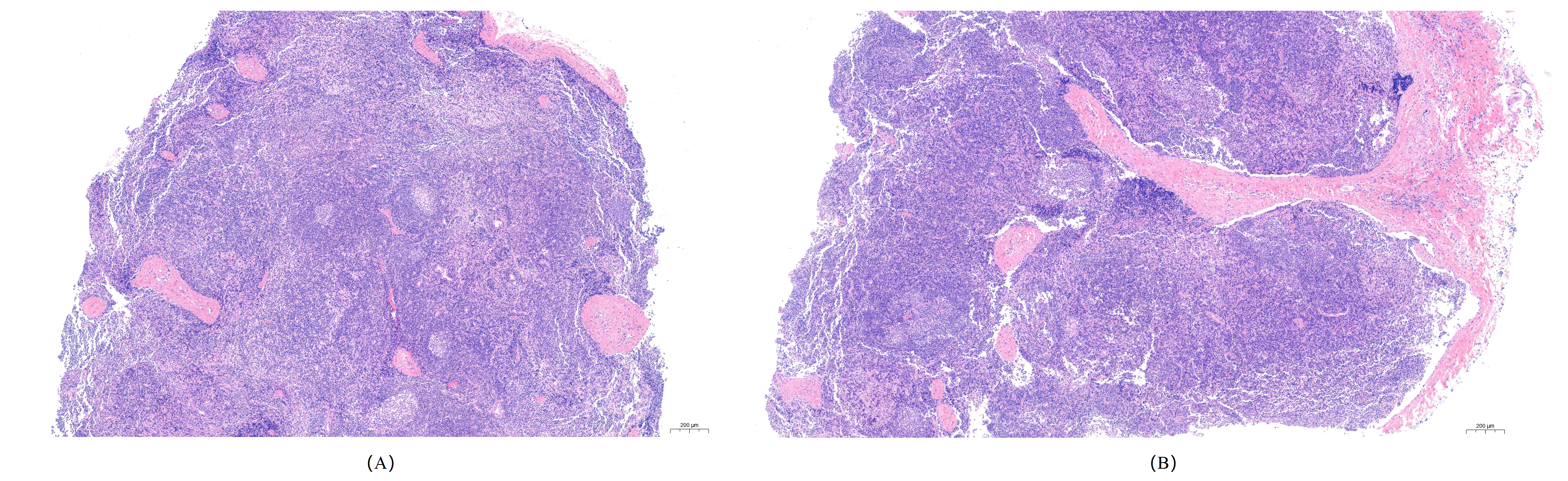

Supplement: Supplementary file 1 — Additional file 1. The histopathology of mandibular lymph nodes at different times. A 0 dpi; B 7 dpi. (H&E, 5×). [file 13567_2023_1147_MOESM1_ESM.tif]
